# Lightweight Knowledge Distillation-Based Transfer Learning Framework for Rolling Bearing Fault Diagnosis

**DOI:** 10.3390/s24061758

**Published:** 2024-03-08

**Authors:** Ruijia Lu, Shuzhi Liu, Zisu Gong, Chengcheng Xu, Zonghe Ma, Yiqi Zhong, Baojian Li

**Affiliations:** School of Physics and Electronic Engineering, Qilu Normal University, Jinan 250200, China; ruijialu01@163.com (R.L.); zisugongsdu@163.com (Z.G.); chengchengxu24@163.com (C.X.); zonghema0508@163.com (Z.M.); yiquzhong2004@163.com (Y.Z.); baojianli03@163.com (B.L.)

**Keywords:** lightweight, knowledge distillation, variational-scale residual networks, multi-kernel domain adaptation approach

## Abstract

Compared to fault diagnosis across operating conditions, the differences in data distribution between devices are more pronounced and better aligned with practical application needs. However, current research on transfer learning inadequately addresses fault diagnosis issues across devices. To better balance the relationship between computational resources and diagnostic accuracy, a knowledge distillation-based lightweight transfer learning framework for rolling bearing diagnosis is proposed in this study. Specifically, a deep teacher–student model based on variable-scale residual networks is constructed to learn domain-invariant features relevant to fault classification within both the source and target domain data. Subsequently, a knowledge distillation framework incorporating a temperature factor is established to transfer fault features learned by the large teacher model in the source domain to the smaller student model, thereby reducing computational and parameter overhead. Finally, a multi-kernel domain adaptation method is employed to capture the feature probability distribution distance of fault characteristics between the source and target domains in Reproducing Kernel Hilbert Space (RKHS), and domain-invariant features are learned by minimizing the distribution distance between them. The effectiveness and applicability of the proposed method in situations of incomplete data across device types were validated through two engineering cases, spanning device models and transitioning from laboratory equipment to real-world operational devices.

## 1. Introduction

With the rapid development of modern industry and science and technology, highly precise and integrated industrial equipment has been widely adopted across various fields. This trend has led to an increased risk of mechanical failures, particularly in rotating components such as bearings [1,2]. However, detecting abnormal noises or subtle faults in bearings can be challenging, making it difficult to mitigate potential risks [3,4]. Minor issues may lead to equipment malfunction or downtime, resulting in economic losses, while more serious failures can pose catastrophic safety hazards [5].

With the rise of deep learning and revolutionary breakthroughs in computer hardware, deep learning-based fault diagnosis algorithms have gained popularity, fueled by the availability of massive datasets [6]. Convolutional neural networks (CNNs) are the most commonly used network in deep learning, achieving excellent results in feature recognition [7]. The local receptive field and weight sharing improve the training speed of CNNs. Deep convolutional networks exhibit outstanding classification performance and have been widely applied in rolling bearing fault diagnosis. Wang et al. [8] proposed an intelligent fault diagnosis method for bearings based on the combination of symmetrical dot pattern representation and a squeeze-excitation convolutional neural network model. Li et al. [9] introduced a novel bearing fault diagnosis model based on ensemble deep neural networks and CNN, where each local network is trained with different datasets to extract diverse features, thereby integrating features with different resolutions for fault identification. Shenfield et al. [10] introduced a new dual-path recurrent neural network (RNN-WDCNN) with a wide one-step kernel and deep CNN path, capable of operating on raw time signals (e.g., vibration data) to diagnose bearing fault data collected from electromechanical drive systems.

However, applying deep learning strategies to rolling bearing fault diagnosis often encounters challenges such as imbalanced data distribution leading to poor generalization of deep diagnostic networks, high memory consumption, and large computational resource utilization by deep models [11,12,13,14]. The emergence of transfer learning provides a fresh and effective approach to address such practical issues, leveraging previously acquired knowledge to assist in learning new knowledge [15]. Shen et al. [16] adjusted the weights between selectively assisted data in the TrAdaBoost algorithm to enhance diagnostic capability, while avoiding negative transfer by judging similarity, thus improving the algorithm accuracy and reducing the computational burden. Liao [17] proposed a transfer network based on dynamic distribution adaptation for cross-domain bearing fault diagnosis. Zhang et al. [18] first extracted features from source and target domain data using CNN, then minimized the probability distribution distance of multi-kernel maximum mean difference and maximized the domain recognition error of the domain classifier to reduce domain distribution differences. Zhou et al. [19] introduced a domain adaptation method, utilizing mixed distance measures to minimize distribution differences between source and target domains, applied to bearing fault diagnosis under different operating conditions. Zhao et al. [20] designed a deep transfer diagnostic method, achieving comprehensive optimization of sample probability distribution distance, model classification error, and domain classification error. Tong et al. [21] proposed a feature transfer learning-based domain adaptation method to address the performance degradation of fault diagnosis models in varying operating condition environments. These transfer methods have demonstrated excellent diagnostic performance under cross-condition scenarios but are challenging to apply to fault diagnosis problems under cross-device scenarios [22,23]. Cross-device scenarios not only involve different operating scenes and environmental changes but also encompass devices of different types with distinct materials, sizes, configurations, or installation methods [24]. These varying factors inevitably lead to more significant distribution differences between the source and target domain data, necessitating research into fault diagnosis methods with better generalization capabilities [25,26].

Knowledge distillation, proposed and popularized by Geoffrey Hinton et al. in 2015 [27], is a technique that can be viewed as a special case of transfer learning. It transfers knowledge from a complete large network (teacher model) to a smaller network (student model) in the form of soft labels, thereby enhancing the accuracy of the network [28,29]. Therefore, this paper proposes a domain-adaptive residual network model based on the knowledge distillation framework. To prevent gradient vanishing as CNNs deepen, a variable-scale residual network is employed to extract domain-invariant fault features from both the source and target domain data. Based on the variable-scale residual network, a knowledge distillation framework is constructed, enabling the student model in the framework to reference soft label information from the teacher model while reducing the model’s size for ease of deployment in industrial scenarios. Domain adaptation is achieved by measuring the difference between the source and target domain data in the feature space using maximum mean discrepancy (MMD), enabling cross-device invariant feature learning.

The main contents of each section of this paper are described as follows: Section 2 introduces the theoretical foundation of the paper, Section 3 details the designed lightweight distillation transfer learning diagnostic model, Section 4 conducts engineering case verification and analysis, and Section 5 provides the conclusions of the paper.

## 2. Theoretical Foundation

### 2.1. Residual Network

**Figure 1:**
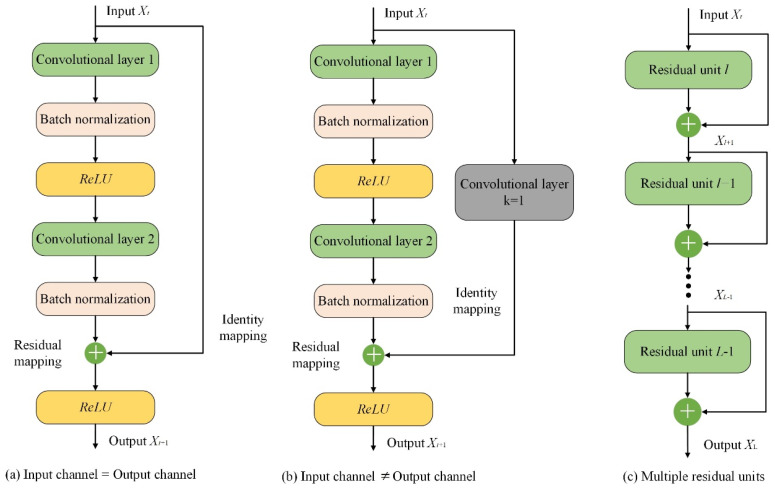
Schematic diagram of ResNet.

The residual network (ResNet) [30] model consists of a series of stacked residual units, each containing two main components: identity mapping and residual mapping. The identity mapping directly connects the input X to the output, while the residual mapping transforms the input through a residual connection (shortcut connection). The principal diagram is shown in Figure 1a, and the output of the residual unit can be calculated as follows:

(1) $$X_{l+1}=X_{l}+F(X_{l})$$

where $X_{l}$ denotes the input data, $X_{l+1}$ denotes the output data, and F() stands for the convolutional operation.

During the convolution operation, the number of channels in the input data $X_{l}$ may differ from that in the output data $X_{l+1}$. In such cases, a convolution with a kernel size of 1 is required to either increase or decrease the dimensionality of the input data, ensuring consistency in the number of channels between the input and output data. This principle is illustrated in Figure 1b, where the output of the residual unit can be calculated as follows:

(2) $$X_{l+1}=Conv_{k=1}(X_{l})+F(X_{l})$$

where $Conv_{k=1}(\cdot )$ represents the convolution operation with a kernel size of 1. For deeper layers *L* in the ResNet model, they can be represented as the sum of any shallow layer *l* and the residual part between two layers. This principle is illustrated in Figure 1c, and the specific output can be calculated using the following equation:

(3) $$X_{L}=X_{l}+\sum_{i=1}^{L-1}F(X_{i})$$

where $\sum_{i=1}^{L-1}F(X_{i})$ represents the sum of the residual mappings of each residual unit. According to the chain rule of derivatives in backpropagation, the gradient of the loss function ε with respect to $X_{l}$ can be expressed as:

(4) $$\frac{\partial \varepsilon }{\partial X_{l}}=\frac{\partial \varepsilon }{\partial X_{L}}\frac{\partial X_{L}}{\partial X_{l}}=\frac{\partial \varepsilon }{\partial X_{L}}\left(1+\frac{\partial }{\partial X_{l}}\sum_{i=1}^{L-1}F(X_{i})\right)$$

Observing Equation (4), it can be seen that regardless of how small the derivative parameters of $\frac{\partial }{\partial X_{l}}\sum_{i=1}^{L-1}F(X_{i})$ are, it ensures that there will be no gradient vanishing during the parameter update of the residual network at this node. This type of residual unit enables better gradient propagation during model training, leading to faster training and convergence speeds.

### 2.2. Knowledge Distillation Model with Soft Labels

**Figure 2:**
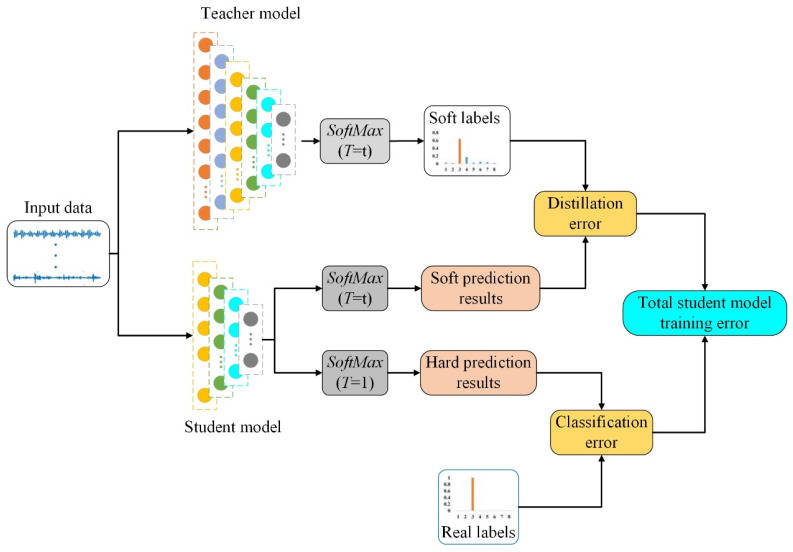
Principle of knowledge distillation.

Knowledge distillation models typically consist of two main stages: the first stage involves the teacher model inferring the training data to obtain soft labels for the classification task, while the second stage entails training the student model using the richer information contained in the soft labels [28]. This process is illustrated in Figure 2. Normally, the model’s prediction results represent the probability predictions for each class in the classification task after passing through the *Softmax* classification layer. However, these probabilities often do not contain information about the similarity between different classes, which can weaken the learned feature information to some extent. Therefore, the teacher model introduces a temperature factor *T* into the *Softmax* function to capture the similarity information between different classes in the classification task, as shown in the following equation:

(5) $$q_{i}^{T}=\frac{\exp(z_{i}/T)}{\sum_{j}\exp(z_{j}/T)}$$

where $z_{i}$ and $z_{j}$ are the inputs to the *Softmax* function, $q_{i}$ represents the predicted probabilities for each class in the classification task, and *T* is the temperature factor. Introducing the temperature factor makes the output probabilities of *Softmax* smoother. When *T* = 1, Equation (5) is equivalent to the traditional *Softmax* classifier.

During the training process of the student model, knowledge distillation introduces the predictions of the teacher model as additional targets while learning the error between the input data and the true sample labels. Generally, the cross-entropy loss function is chosen as the loss calculation function between the model’s test probability values and the true labels. Then, the distilled loss $C_{distill}$ between the teacher model and the student model, as well as the loss $C_{class}$ between the predictions of the student model and the true labels, are represented by Equation (6) and Equation (7), respectively.

(6) $$L_{soft}=-\sum_{j}^{N}p_{j}^{T}\log(q_{j}^{T})$$

(7) $$L_{hard}=-\sum_{j}^{N}c_{j}\log(q_{j}^{1})$$

where $p_{j}^{T}$ and $q_{j}^{T}$, respectively, denote the predicted probability distributions of the teacher and student models after distillation with temperature factor *T*, $c_{j}$ represents the true labels of the classification task, and $q_{j}^{1}$ represents the predicted probability distribution of the student model when the temperature factor *T* is 1, also known as the hard label prediction of the student model. The loss of the entire knowledge distillation model consists of two parts, denoted by $L_{soft}$ and $L_{hard}$, respectively, as shown in Equation (8):

(8) $$L=\alpha L_{soft}+(1-\alpha )L_{hard}$$

where α represents the weighting of the model loss considering soft labels.

### 2.3. Maximum Mean Discrepancy

MMD projects input data onto the Reproducing Kernel Hilbert Space (RKHS) by defining a kernel function, transforming complex relationships that are linearly inseparable in low-dimensional space into linear relationships in high-dimensional space, thereby describing the statistical properties of the data [31]. The definition of the distance between two probability distributions in RKHS is as follows:

(9) $$MMD(X,Y)={\left\|\frac{1}{n}\sum_{i=1}^{n}\phi (x_{i})-\frac{1}{m}\sum_{j=1}^{m}\phi (y_{i})\right\|}_{H}^{2}$$

where *X* and *Y* represent the source domain and target domain datasets, respectively, *H* denotes the measurement of data mapped to RKHS, and if the MMD value tends to zero, it indicates that the two probability distributions are similar. Expanding Equation (9) yields the following:

(10) $$MMD(X,Y)=\left\|\frac{1}{n^{2}}\sum_{i}^{n}\sum_{i^{\prime }}^{n}\phi (x_{i})\phi (x_{i}^{\prime })-\frac{1}{nm}\sum_{i}^{n}\sum_{j}^{m}\phi (x_{i})\phi (y_{j})+\frac{1}{m^{2}}\sum_{j}^{m}\sum_{j^{\prime }}^{m}\phi (y_{j})\phi (y_{j}^{\prime })\right\|$$

where the inner product calculation of two vectors $\phi (x_{i})\phi (x_{i}^{\prime })$ can implicitly map data to a high-dimensional feature space through the kernel function k(⋅). Therefore, MMD can also be expressed as follows:

(11) $$MMD(X,Y)=\left\|\frac{1}{n^{2}}\sum_{i}^{n}\sum_{i^{\prime }}^{n}k(x_{i},x_{i}^{\prime })-\frac{1}{nm}\sum_{i}^{n}\sum_{j}^{m}k(x_{i},y_{j})+\frac{1}{m^{2}}\sum_{j}^{m}\sum_{j^{\prime }}^{m}k(y_{j},y_{j}^{\prime })\right\|$$

The kernel function k(⋅) is typically a Gaussian kernel function, as shown in Equation (12).

(12) $$k(x,x^{\prime })=\exp\left(-\frac{{\left\|x-x^{\prime }\right\|}^{2}}{2\sigma^{2}}\right)$$

where $x^{\prime }$ is the kernel function center, ${\left\|x-x^{\prime }\right\|}^{2}$ represents the Euclidean distance between vector x and vector $x^{\prime }$, and σ represents the kernel function width, which also controls the range of influence of the Gaussian kernel function. When σ is relatively large, changes in ${\left\|x-x^{\prime }\right\|}^{2}$ have a small impact on the kernel function, indicating that changes in $k(x,x^{\prime })$ are relatively “smooth”; when σ is relatively small, changes in ${\left\|x-x^{\prime }\right\|}^{2}$ have a greater impact on the kernel function, indicating that changes in $k(x,x^{\prime })$ are relatively “sharp”.

## 3. Lightweight Distillation Transfer Learning Diagnostic Model

**Figure 3:**
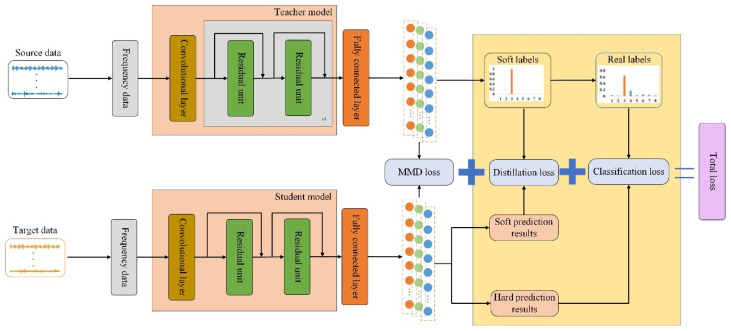
Network structure of KD-ResNet-DA.

This paper fully considers the advantages and limitations of deep and shallow deep learning networks. Based on the techniques of knowledge distillation lightweight models and feature-level domain adaptation transfer, it investigates model compression while ensuring the performance of deep learning models. Addressing the task of fault diagnosis across devices with incomplete inter-class data, this paper proposes a knowledge distillation-based residual network with domain adaptation (KD-ResNet-DA). This method utilizes a variable-scale ResNet model to extract domain-invariant features from the source domain data. Employing the framework of knowledge distillation, it transfers the fault features extracted by the deep teacher model from the source domain to the smaller-volume student model, achieving the extraction of fault features in the target domain. Furthermore, it minimizes the probability distribution distance of fault features between the source and target domains, facilitating domain-invariant feature learning across devices. The structure of the KD-ResNet-DA network model is illustrated in Figure 3, comprising primarily the knowledge distillation framework and feature-level domain adaptation transfer. The knowledge distillation framework enables the student model to reference the internally invariant features learned by the teacher model during training. Meanwhile, domain adaptation transfer learns mutually invariant features of probability distribution between the source and target domain data from the feature level. The overall objective function of the model consists of three parts: the distillation error $L_{distill}$ from the knowledge distillation framework, the classification error $L_{class}$ of the student model, and the probability distribution distance loss $L_{MMD}$ at the feature level. The specific methods for obtaining each loss will be discussed in the following two sections.

### 3.1. Acquisition of Knowledge Distillation Framework Error

The knowledge distillation framework considering soft labels mainly consists of a deep variable-scale ResNet teacher model and a shallow ResNet student model. This framework relies on the teacher model to extract domain-invariant features relevant to fault classification tasks from the source domain data and transfer the learned fault classification information to the smaller-volume student model at the classification layer. The specific steps are as follows:

(1) Input source domain data into the deep variable-scale ResNet teacher model: The source domain dataset $X^{Source}={\left\{x^{i},y^{i}\right\}}_{i=1}^{N}\in {ℜ}^{1\times D}$ is input into the deep variable-scale ResNet teacher model. Initially, convolutional layers with larger kernel sizes capture coarse-grained features in a wide frequency band of the frequency domain signals. Then, the variable-scale ResNet progressively converts coarse-grained features into fine-grained fault features, with each residual unit adding a convolutional layer with a kernel size of 1 to reduce the depth of intermediate feature matrices and decrease model parameters. This process yields domain-invariant features $embedding_{II}^{T}$ for the source domain data.
(2) Pretrain the teacher model and infer with *Softmax* classifier: The teacher model is pretrained via backpropagation, and the pretrained variable-scale ResNet teacher model performs inference. By introducing the temperature factor *T* in the *Softmax* classifier as in Equation (5), the probabilities of each sample belonging to each fault class in the classification layer are obtained, i.e., soft labels $Label_{soft}$.
(3) Input target domain data into the shallow student model: The target domain dataset $X^{\mathrm{Target}}={\left\{x^{j},y^{j}\right\}}_{j=1}^{N}\in {ℜ}^{1\times D}$ is input into the shallow student model with fewer parameters, and the features $embedding^{S}$ of the target domain data are obtained.
(4) Calculate distillation error between student model’s soft predictions and soft labels: The distillation error $L_{distill}$ between the soft predictions ${\mathrm{Pre}}_{soft}$ of the student model and the soft labels $Label_{soft}$ is computed using Equation (6).
(5) Calculate classification error between student model’s hard predictions and true sample labels: The classification error $L_{class}$ between the hard predictions ${\mathrm{Pre}}_{hard}$ of the student model and the true sample labels ${\mathrm{Label}}_{hard}$ is calculated using Equation (7).
(6) Utilize overall knowledge distillation framework loss as training objective for student model: The overall loss, composed of distillation error $L_{distill}$ and classification error $L_{class}$, serves as the training objective for the student model, enabling it to gradually approach the outstanding fault classification performance of the teacher model.

### 3.2. Domain Adaptation Loss Acquisition Based on MMD

In the aforementioned knowledge distillation framework, domain-invariant features $embedding_{II}^{T}$ of the source domain data and features $embedding^{S}$ of the target domain data are extracted at the feature level. The probability distribution distance $L_{MMD}$ between the two is then computed in the RKHS using Equation (11). This distance is learned as more diverse domain-invariant features are encouraged through regularization. In Equation (11), the value σ in the Gaussian kernel function k(⋅) represents the width of the kernel function, controlling the range of influence of $embedding_{II}^{T}$ and $embedding^{S}$ on the kernel function, i.e., the smoothness of the Gaussian kernel function. Considering the potential variation in feature distributions across different cross-device fault diagnosis tasks, this study selects multiple values of σ to enhance the flexibility of domain adaptation. By selecting multiple different values of σ, the model can adapt to the diverse data features present in different cross-device scenarios, thus making it more suitable for a variety of fault classification tasks.

### 3.3. Training Procedure of KD-ResNet-DA

The proposed method represents a novel integration of the knowledge distillation framework with domain adaptation at the feature level, offering a comprehensive approach to address the challenges posed by cross-domain data incompleteness. By leveraging the strengths of both techniques, the proposed approach facilitates the seamless transfer of domain-invariant features gleaned from the teacher model trained on the source domain data to the student model. This transfer ensures that the student model can effectively capture and utilize essential information without being hindered by domain discrepancies. Moreover, our method goes beyond traditional knowledge distillation by incorporating domain adaptation mechanisms to bridge the gap between the source and target domains. Specifically, it exploits multi-kernel MMD to discern domain-invariant features between the source and target domain data. This process enhances the adaptability of the model to diverse data distributions encountered in real-world scenarios, thereby improving its robustness and generalization capability. The ultimate objective function of the proposed KD-ResNet-DA method encapsulates the essence of these strategies, aiming to minimize the distillation error, classification error, and probability distribution distance simultaneously. The distillation error quantifies the discrepancy between the soft predictions of the student model and the soft labels provided by the teacher model, facilitating the transfer of knowledge effectively. The classification error measures the disparity between the hard predictions of the student model and the ground truth labels, ensuring accurate diagnostic outcomes. Additionally, the probability distribution distance captures the dissimilarity between the probability distributions of the source and target domain data, guiding the model towards learning domain-invariant representations.

In essence, the proposed KD-ResNet-DA method offers a synergistic fusion of knowledge distillation and domain adaptation techniques, underpinned by a comprehensive objective function that optimizes model performance across domains. This holistic approach not only enhances the diagnostic accuracy and efficiency but also lays the groundwork for advancing intelligent fault diagnosis in diverse industrial settings. The ultimate objective function $L_{final}$ of the proposed KD-ResNet-DA method consists of distillation error $L_{distill}$, classification error $L_{class}$, and probability distribution distance $L_{MMD}$, which can be defined as follows:

(13) $$L_{final}=\alpha L_{distill}+(1-\alpha )L_{class}+L_{MMD}$$

where α represents the relative weight balancing between the distillation error of the teacher model and the classification error of the student model.

## 4. Experimental Validation and Analysis

To verify the effectiveness and applicability of the proposed model under the scenario of cross-device situations and incomplete inter-class data, two engineering case studies of different degrees of cross-device variations were conducted. One involves the verification case of different bearing models, while the other spans from laboratory bearings to real-world operational bearings, further confirming the versatility of the proposed algorithm.

### 4.1. Experimental Samples and Network Structure Parameters

The network structures of the deep variable-scale ResNet teacher model, shallow ResNet student model, and fault classification module in the proposed KD-ResNet-DA method are outlined in Table 1, Table 2, and Table 3, respectively. The teacher model has a floating-point operation count (FLOP) of 225,609,728.0 and 2,274,496.0 parameters, while the student model has FLOPs of 34,932,736.0 and 268,864.0 parameters. Compared to the teacher model, the student model reduces the computational complexity by one-sixth and the parameter count by one-eighth.

To validate the proposed model under cross-device scenarios with incomplete inter-class data, incomplete inter-class sample sets were constructed for each validation case. Each set consists of 300 healthy state samples, each composed of 2048 sampling points, with only 5 fault samples selected from each of the remaining fault states. The experimental parameters, including learning rate, training iterations, etc., are listed in Table 4.

### 4.2. Cross-Device Case Validation

#### 4.2.1. Dataset Illustration

**Figure 4:**
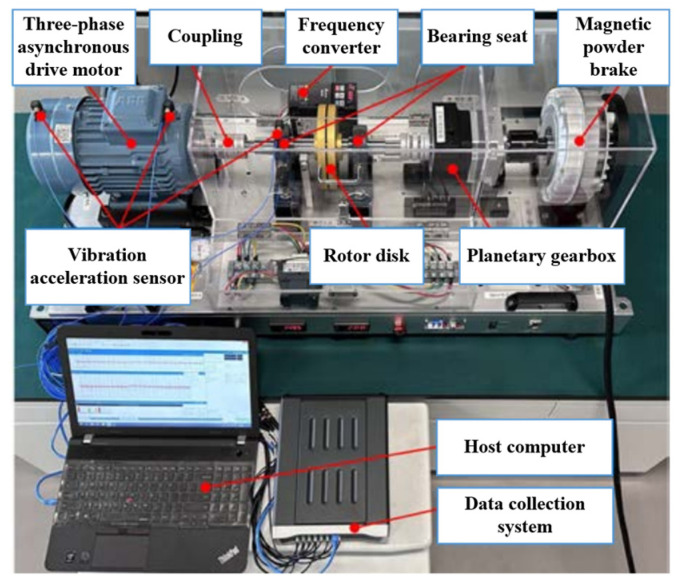
Homemade rotating machinery fault test platform.

In this case study, two different models of bearings are selected for experimental validation. The source domain dataset A is derived from the publicly available bearing fault dataset from Case Western Reserve University (CWRU) [32], featuring SKF6205 deep groove ball bearings with a fault size of 0.5334 mm operating at 1772 r/min and sampled at 12 kHz. The target domain dataset B originates from the bearing seat vibration signal dataset from Qilu Normal University (QLNU), as illustrated in Figure 4. The selected model is UCPH206 ball bearings with a fault size of 1 mm operating at 900 r/min and sampled at 51.2 kHz. The selection of vibration acceleration sensor type is chosen as an accelerometer sensor. The motor power ranges from 0.5 kW to 5 kW, and the maximum load for the electric brake is 100 Nm. The health status labels for both datasets are presented in Table 5.

#### 4.2.2. Experimental Results and Discussion

**Figure 5:**
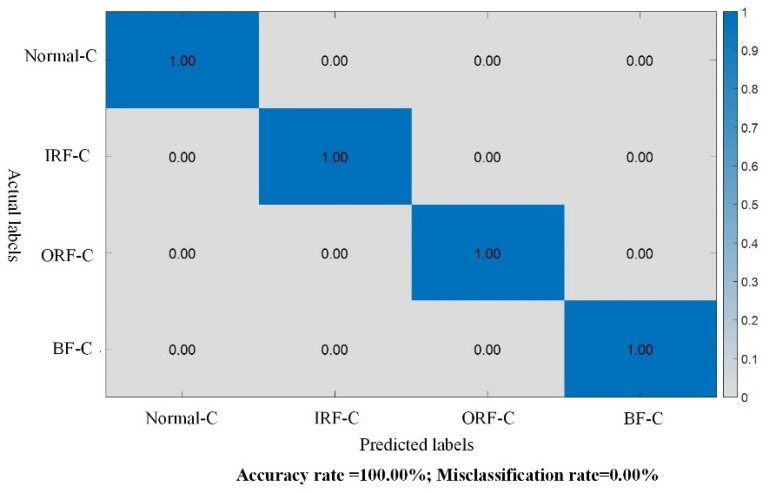
Test results of the teacher model in Case 1.

Initially, the source domain dataset A is fed into the knowledge distillation framework of the proposed KD-ResNet-DA method. The variable-scale ResNet teacher model is utilized to extract fault features from the source domain dataset for training. The testing results on the source domain dataset are illustrated in Figure 5. It can be observed that the accuracy of the teacher model in the KD-ResNet-DA method is 100.00%.

**Figure 6:**
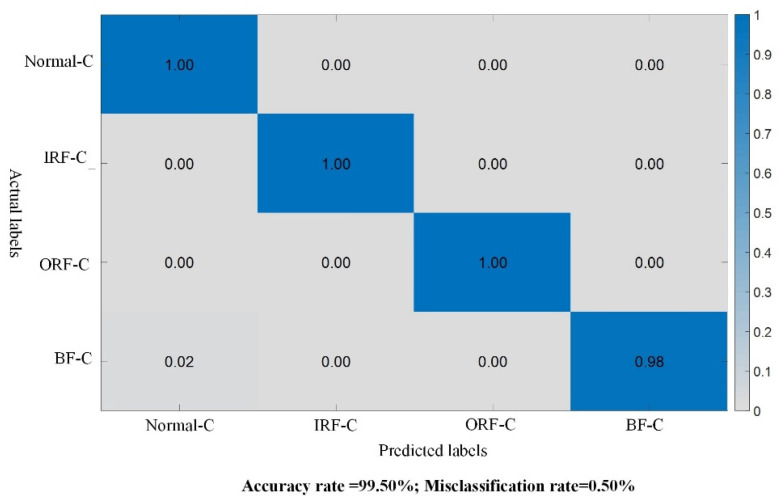
Test results of the student model in Case 1.

Subsequently, based on the fault soft labels obtained from the teacher model, the constructed QLNU bearing target domain dataset with incomplete inter-class data is inputted into the student model of the proposed KD-ResNet-DA method for training and testing. The experimental results are depicted in Figure 6. It is evident that the accuracy of the student model in the target domain reaches 99.50%. This preliminary validation confirms the effectiveness of the proposed model under the scenario of cross-device incomplete inter-class data.

### 4.3. Cross-Device Case Study: From Laboratory Bearings to Real-World Bearings

#### 4.3.1. Dataset Illustration

In this case study, experiments are conducted to validate the proposed method using laboratory bearings and real-world bearings installed in motors. The source domain dataset A comprises vibration signal samples from faulty bearing seats collected from the rotating machinery fault simulation test bench shown in Figure 4. The selected bearing model is a UCPH206 ball bearing with a fault size of 1 mm rotating at 900 r/min and sampled at 51.2 kHz. The target domain dataset B consists of vibration signal samples from motors with faulty bearings collected from the same rotating machinery fault simulation test bench depicted in Figure 4. The selected bearing model is a 6205 deep-groove ball bearing with a fault size of 3 mm rotating at 1500 r/min and sampled at 51.2 kHz. The health status labels for both datasets are presented in Table 6.

#### 4.3.2. Discussion of Experimental Results

**Figure 7:**
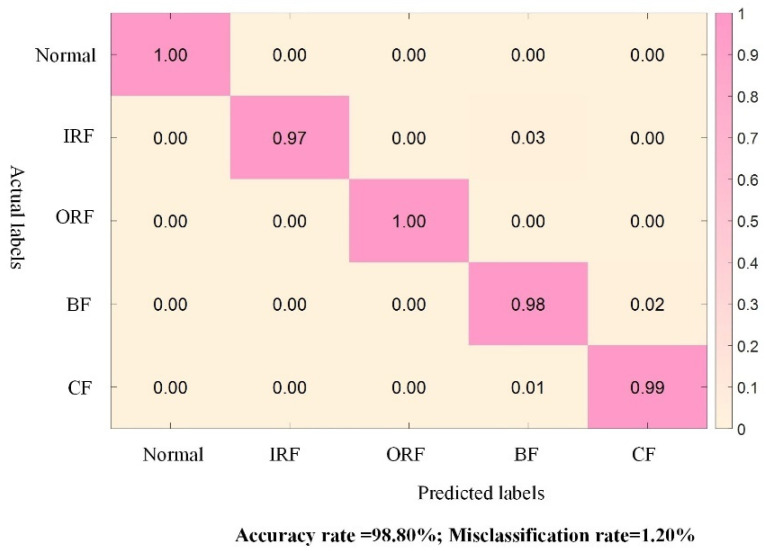
Test results of the teacher model in Case 2.

To validate the superiority of the proposed KD-ResNet-DA method and explore the contributions of the teacher model considering soft labels, domain adaptation loss, and multi-scale ResNet, three sets of ablation experiments were designed for comparative analysis. The descriptions of each experimental group are as follows: 1. ResNet-DA: The distillation loss provided by the teacher model in the target loss function is removed. 2. KD-ResNet: The loss of probability distribution distance at the feature level is removed. 3. KD-CNN-DA: The shortcut connection in the multi-scale residual network model is removed, and a one-dimensional convolution with a kernel size of 3 is used to replace the original multi-scale convolution in the model. To ensure fairness in the ablation experiments, the network parameters of the three comparison algorithms are kept consistent with KD-ResNet-DA (Figure 7).

**Figure 8:**
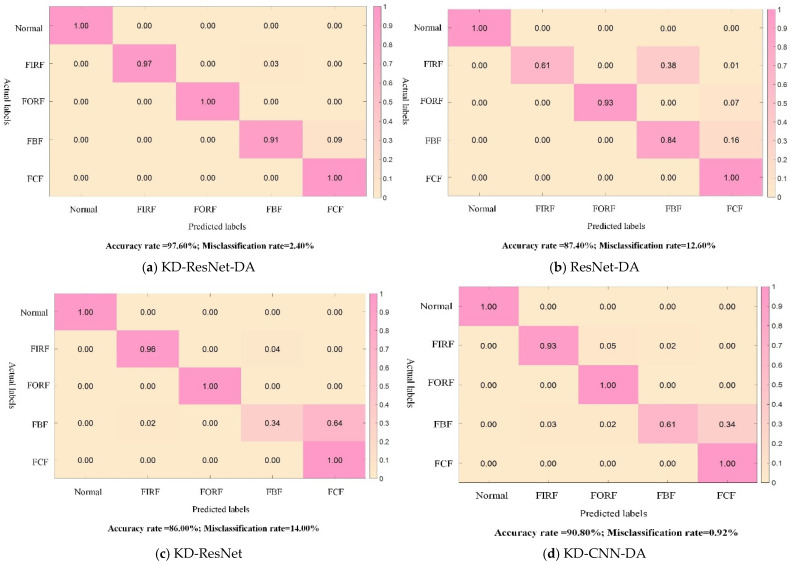
Confusion matrix of student model ablation experiment results.

Next, based on the fault soft labels obtained from the teacher model, the established target domain inter-class incomplete dataset is input into the student models of the KD-ResNet-DA method and the three comparison methods for training and testing. The experimental results of KD-ResNet-DA, ResNet-DA, KD-ResNet, and KD-CNN-DA in the ablation experiments are shown in Figure 8. The accuracies of KD-ResNet-DA, ResNet-DA, KD-ResNet, and KD-CNN-DA are 97.60%, 87.40%, 86.00%, and 90.80%, respectively, with only the proposed KD-ResNet-DA method achieving an accuracy higher than 95.00%. This preliminarily proves the effectiveness of the KD-ResNet-DA method in the presence of cross-device inter-class incomplete data and validates the contributions of the distillation loss provided by the teacher model, the loss of probability distribution distance at the feature level, and the multi-scale residual network model proposed in this study.

**Figure 9:**
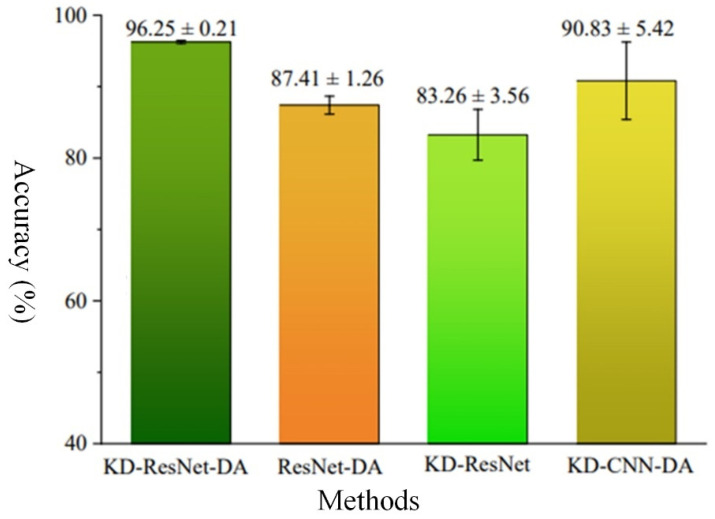
The ablation experiment results of student model.

**Figure 10:**
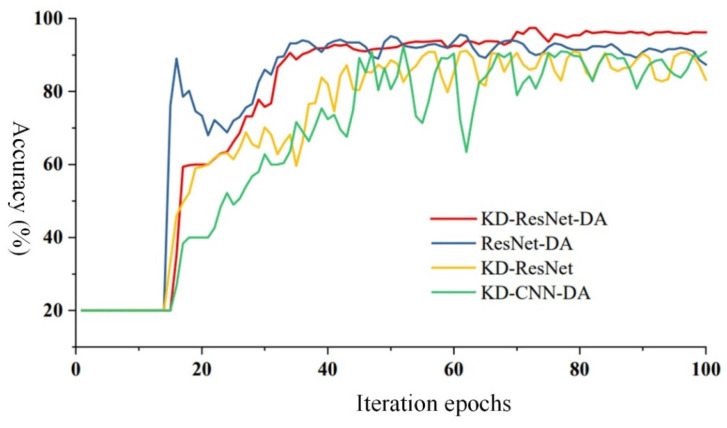
The comparison of accuracy in the ablation experiments.

Figure 9 presents a bar chart of “mean accuracy ± standard deviation” of the various algorithms in the ablation experiments, illustrating the accuracy and robustness of each algorithm. It can be observed that the proposed KD-ResNet-DA method exhibits the highest diagnostic accuracy, maintaining an accuracy of over 95.00% in the presence of cross-device inter-class incomplete data. The remaining three methods are ranked by accuracy as KD-CNN-DA, ResNet-DA, and KD-ResNet. The proposed KD-ResNet-DA method also demonstrates the highest diagnostic robustness, with a standard deviation of around 0.20% even in the presence of cross-device inter-class incomplete data, while the remaining three methods are ranked by robustness as ResNet-DA, KD-ResNet, and KD-CNN-DA. Combining with the accuracy curve comparison chart of the four methods in Figure 10, further analysis of the convergence speed and stability of each method can be conducted. It can be observed that all four methods begin to converge around the 20th iteration. Among them, the proposed KD-ResNet-DA method exhibits the best convergence speed and stability. The ResNet-DA method, which removes the distillation loss of the teacher model, quickly reaches around 90% accuracy in the early iterations, but then gradually stabilizes after a significant drop in accuracy. This indicates that the teacher model in the knowledge distillation framework provides more stable and essential fault information for training the student model on target domain data. Both the KD-ResNet method, which removes the loss of probability distribution distance at the feature level, and the KD-CNN-DA method, which removes the multi-scale residual network model, exhibit varying degrees of fluctuations during training, especially KD-CNN-DA’s convergence is relatively slow and the fluctuation amplitude is larger. This suggests that the feature-level domain adaptation method can extract domain-invariant features between source and target domains, thereby improving the model’s robustness, and the shortcut connections in the ResNet model can excavate more domain-invariant features from the source and target domain data, respectively.

#### 4.3.3. Comparison with Other Classical Algorithms

Building upon the analysis of the aforementioned cross-condition experimental results, a comparison is made with mainstream advanced algorithms in the recent literature regarding cross-device scenarios with incomplete inter-class data. These include data augmentation methods based on SMOTE and GAN, domain adaptation methods based on MMD and CORAL, and domain-adversarial neural networks (DANN). The comparative diagnostic accuracy results are presented in Table 7. Notably, the proposed KD-ResNet-DA method achieves an average accuracy of 96.25%, significantly higher than the other algorithms listed in the table. This further underscores the effectiveness and superiority of the proposed method in scenarios involving incomplete inter-class data across different devices.

#### 4.3.4. Impact of Distillation Loss Weight on Diagnostic Results

In the knowledge distillation model, parameter α is used to balance the relative importance between the teacher and student model predictions, serving as a crucial hyperparameter in the proposed method’s target loss function. Typically ranging between 0 and 1, it denotes the proportion of importance between the teacher and student models. By adjusting the value of α, the balance between the two models can be fine-tuned to better transfer the knowledge from the teacher model to the student model. When α approaches 0, more weight is assigned to the student model, allowing it to focus more on the hard labels relevant to the fault task during training. Conversely, when α approaches 1, more weight is given to the teacher model, enabling the student model to pay greater attention to the soft labels provided by the teacher model, thereby achieving smoother and more generalized prediction results.

**Figure 11:**
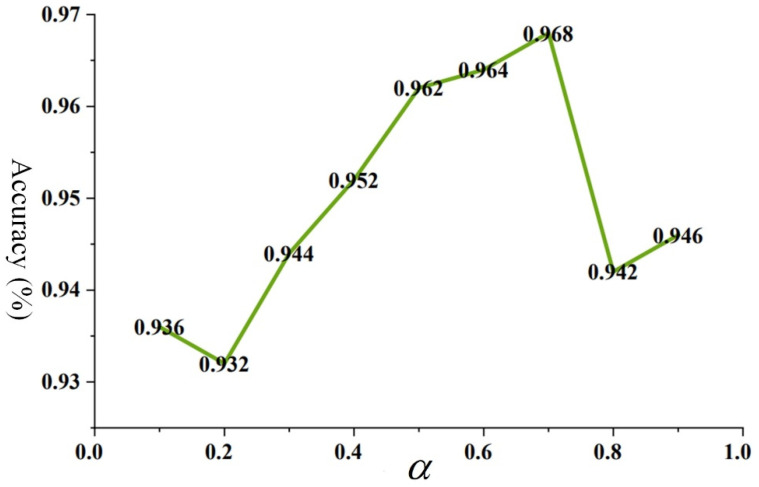
The impact of value on KD-ResNet-DA diagnostic results.

Figure 11 illustrates the impact of different α values on the diagnostic results of KD-ResNet-DA. Generally, the choice of α needs to be adjusted according to the specific task and dataset, as different settings may yield different effects. Therefore, in this case, experiments were conducted to investigate the influence of the distillation loss weight on the diagnostic results. As shown in Figure 11, the accuracy increases as the value of α increases. The KD-ResNet-DA model achieves optimal accuracy when α values are 0.6 and 0.7. However, as it approaches 1, the model’s accuracy rapidly decreases. This further confirms that both classification loss and distillation loss contribute to the model’s performance gains.

## 5. Conclusions

The intelligent fault diagnosis method proposed in this study, based on knowledge distillation and domain adaptation residual networks, demonstrates a scientific rationale and practical utility. By addressing the issue of incomplete inter-class data in cross-device scenarios, it overcomes the contradiction between diagnostic accuracy and computational resources during model deployment. Training the teacher model using frequency domain data and introducing soft labels with a temperature factor enables the extraction of domain-invariant features from the source domain data, providing valuable information for the model. Subsequently, by computing the distillation error and classification error, along with measuring the probability distribution distance loss using a multi-kernel Gaussian kernel function, high-performance fault diagnosis can be achieved while maintaining a smaller model size.

Although this study operates under the premise of identical sample labels between the source and target domains, this assumption does not hinder the effectiveness of the proposed method in practical applications. In future research, the team will further focus on addressing open-set fault diagnosis issues to enhance the applicability and generality of the method, thereby contributing significant scientific value to the advancement of intelligent fault diagnosis across devices.

## Figures and Tables

**Table 1:** The network structure of KD-ResNet-DA teacher model.

Serial Number	Layer Type	Kernel Size	Stride	Padding	Output
1	Convolution	7	2	3	[batch_size, 64, 512]
	Pooling	3	2	1	[batch_size, 64, 256]
Residual unit 1	Convolution	1	1	0	[batch_size, 256, 256]
	Convolution	5	1	2	
Residual unit 2	Convolution	1	1	0	[batch_size, 256, 256]
	Convolution	5	1	2	
Residual unit 3	Convolution	1	1	0	[batch_size, 512, 128]
	Convolution	3	1	1	
Residual unit 4	Convolution	1	1	0	[batch_size, 512, 128]
	Convolution	3	1	1	
Residual unit 5	Convolution	1	1	0	[batch_size, 1024, 64]
	Convolution	1	1	0	
Residual unit 6	Convolution	1	1	0	[batch_size, 1024, 64]
	Convolution	1	1	0	

**Table 2:** The network structure of KD-ResNet-DA student model.

Serial Number	Layer Type	Kernel Size	Stride	Padding	Output
1	Convolution	7	2	3	[batch_size, 64, 512]
	Pooling	3	2	1	[batch_size, 64, 256]
Residual unit 1	Convolution	1	1	0	[batch_size, 512, 128]
	Convolution	3	1	1	
Residual unit 2	Convolution	1	1	0	[batch_size, 1024, 64]
	Convolution	3	1	1	

**Table 3:** The network structure of fault classification module.

Name	Type	Number of Neurons	Output
Feature Reduction	Adaptive average pooling	-	[batch_size, 1024, 2]
	Fully connected	2048–1024	[batch_size, 1024]
	Fully connected	1024–512	[batch_size, 512]
Fault Classification	*Softmax*	-	[batch_size, *C*]

**Table 4:** Experimental parameter settings.

Parameters	Values
Learning rate	1 × 10^−4^
Number of training iterations	100
Batch size for training	64
Gaussian kernel σ value set	[0.25, 0.5, 1, 2, 4]
Temperature factor *T*	2

**Table 5:** Health status labels for cross-model case study.

Fault Mode	Label	Fault Mode
Normal-C	0	Normal-Q
Inner race fault (IRF-C)	1	Inner race fault (IRF-Q)
Outer race fault (ORF-C)	2	Outer race fault (ORF-Q)
Ball fault (BF-C)	3	Ball fault (BF-Q)

**Table 6:** Health status labels for cross-device case study.

Fault Mode	Label	Fault Mode
Normal	0	Normal
IRF	1	Front inner race fault (FIRF)
ORF	2	Front outer race fault (FORF)
BF	3	Front ball fault (FBF)
Cage fault (CF)	4	Front cage fault (FCF)

**Table 7:** Comparative results of the proposed method with other classical algorithms.

Diagnosis Methods	Accuracy
SMOTE	64.18%
GAN	76.62%
CORAL	82.84%
DANN	89.38%
KD-ResNet-DA	96.25%

## Data Availability

The data used to support the findings of this study are available from the corresponding author upon request.
